# Real-Time Plasmonic Strain Sensors Based on Surface Relief Diffraction Gratings

**DOI:** 10.3390/mi15070863

**Published:** 2024-06-30

**Authors:** Yazan Bdour, Ribal Georges Sabat

**Affiliations:** Department of Physics and Space Science, Royal Military College of Canada, STN Forces, P.O. Box 17000, Kingston, ON K7K 7B4, Canada; georges.sabat@rmc.ca

**Keywords:** strain sensor, PDMS gratings, stretchable flexible nanostructures, plasmonics

## Abstract

Large-scale diffraction gratings were fabricated in surface relief on azobenzene thin films and transferred to flexible PDMS substrates using soft lift-off lithography. The PDMS gratings were strained along the grating vector axis and the resulting surface topography was analyzed using diffraction angle measurements, AFM imagery and surface plasmon resonance (SPR) spectra. All measurement methods exhibited a linear response in strain indicating the useability of these sensors in real-world applications. For SPR-based strain sensing, an increasing pitch and a decreasing modulation depth were observed with increasing strain. The SPR peak shifted by ~1.0 nm wavelength and the SPR intensity decreased by ~0.3 a.u. per percentage of applied strain. The tested PDMS samples retained their integrity even after multiple cycles of stretching and relaxation, making them a suitable strain sensor.

## 1. Introduction

Surface plasmon resonance (SPR) is an optical phenomenon characterized by the collective oscillation of free electrons at a dielectric–metal interface [1]. Its applications span various fields, such as enhanced optical transmission, optical waveguides and enhanced light generation [2,3,4,5]. Notably, it is used within sensing and spectroscopic platforms, due to its real-time, accurate and reactive response [6,7]. SPR signals are effectively generated and propagated utilizing various nanostructured surfaces ranging from nanohole arrays, nano-slits, gratings and unique metasurfaces [8,9,10,11]. Fabrication techniques for these nanostructures encompass different methodologies such as electron beam lithography and focused ion beam milling, as well as solution-based self-assembly [3,12,13]. Recently, soft lithography garnered particular attention in its capability to produce complex nanostructures with elastomeric substrates [14,15]. This combination of SPR sensing with elastomeric materials holds promise, offering a compelling avenue for flexible and stretchable substrates in real-time strain-sensing applications [16,17].

In our previous work, we demonstrated the all-optical fabrication of various surface relief nanostructures in azobenzene thin films using various laser beam manipulation techniques. More precisely, laser interference patterns generated from optical elements enabled the fabrication of linear surface relief gratings (SRGs), crossed surface relief gratings (CSRGs), concentric circular gratings, and other unique metasurfaces [18,19,20]. These optical nanostructures can be subsequently transferred from the azobenzene thin film to an elastomeric substrate, facilitating their utilization in applications not limited to the physical and chemical properties of the azobenzene film.

Gratings-based SPR sensors have gained popularity due to their unique plasmonic response [21,22]. The spectral response resulting from the SPR generation via the gratings is easily captured and monitored as it directly appears in the reflected and the transmitted light spectra across the substrate. Furthermore, the plasmon response is tunable through the grating’s topology, including depth, pitch, and shape [23]. The integration of gratings into flexible and stretchable substrates presents a promising avenue for developing strain-sensitive platforms. As the gratings deform with the substrate, they induce changes in the SPR response, thereby facilitating real-time strain-sensing capabilities. Previous studies have demonstrated the fabrication of chirped pitch gratings on elastomers through nonuniform buckling, enabling the generation of plasmonic signals [24]. Stretchable and moldable strain sensors hold pivotal roles across diverse fields, including healthcare management, artificial intelligence and the development of wearable products.

In this study, we investigate the enhanced optical transmission resulting from the plasmonic response of polydimethylsiloxane (PDMS)-based gratings fabricated through template lift-off soft lithography. Topographical imaging techniques were employed to track the evolution of the grating pattern under varying levels of strain, allowing the development of a detailed pitch and depth profile correlated with the applied strain. Additionally, we analyze the plasmonic response to characterize the sensitivity of the gratings to strain changes. In comparison to previous research, our work uniquely combines precise soft lithography techniques with plasmonic response analysis to track and correlate the evolution of the change in the pattern under varying levels of strain. Our findings demonstrate a significant advancement over prior studies by establishing the feasibility of using these gratings as highly sensitive strain sensors capable of detecting subtle variations in applied strain on the substrate. This research lays the foundation for the development of more complex structures and metasurfaces on elastomeric substrates. Thus, our work offers promising avenues for future research in the field of flexible and stretchable sensing devices, with potential applications spanning from healthcare to wearable technology.

## 2. Materials and Methods

The fabrication process of PDMS nanostructures employed a soft lithography lift-off method. Disperse Red-1 Azobenzene molecular glass (gDR1) powder was synthesized according to a previously described process [25]. A 3% weight solution of azobenzene dissolved in dichloromethane (DCM) is deposited and spin-coated at 1000 RPM onto a clean Corning 0215 soda lime microscope glass slide to achieve a 300 nm thick azobenzene thin film, as measured using a DektakXT stylus surface profiler (Bruker, San Jose, CA, USA). Subsequently, the film is dried at 80 °C for 10 min to ensure the removal of any trapped solvent within the surface.

The optical nanostructures are then inscribed onto the sample surface using a continuous wave Gaussian laser beam emitted from a 532 nm diode-pumped solid-state laser (Verdi V6, Coherent, Santa Clara, CA, USA). This laser beam is passed through a spatial filter and is made circularly polarized using a quarter-wave plate. It is then expanded, collimated, and passed through an iris to control its size to a circle approximately 2 cm in diameter. For the fabrication of the gratings, a Lloyd mirror interferometer is used to inscribe gratings onto the Azobenzene thin film with a randomly chosen pitch of 700 nm, although a range of other grating pitches could have been equally used.

Next, the nanostructures are coated with a 10 nm layer of gold using a Quorum sputter coater (Quorum, Laughton, UK). These gold-coated structures are then transferred onto a transparent epoxy thin film using a UV nanoimprint soft lithography technique [26]. The UV epoxy (Millipore Sigma Canada, Oakville, ON, Canada) is sandwiched between a glass slide and the gold-coated nanostructures. After curing for 2 min, the epoxy is peeled off, transferring the nanostructures onto the epoxy. Any remaining metal layer is etched away with a chemical metal etchant, resulting in an epoxy-based nanostructure mold suitable for the consistent transfer of nanostructures into PDMS.

In a similar process, a 10:1 mixture of SYLGARD 184 PDMS to curing agent is placed between the epoxy nanostructure mold and a glass slide with 1 mm thick spacers. Following curing at 70 °C for 6 h, the PDMS is peeled off to transfer the nanostructures into it.

**Figure 1:**
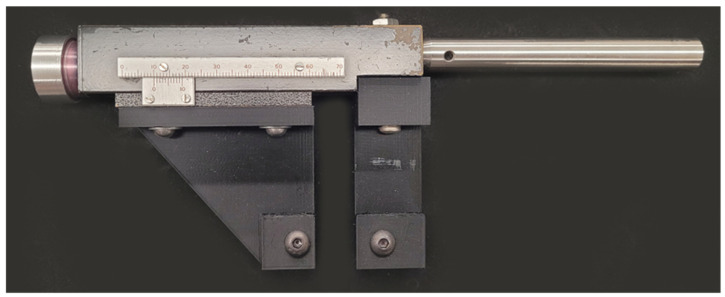
Image the in-house-built fixture used to apply linear strain to the nanostructured PDMS sample. The fixture features a side-mounted knob for controlled movement of the clamps, enabling precise incremental stretching. A linear metric scale with a Vernier scale is used to accurately measure the separation distance as the sample is strained. Additionally, the fixture includes an optical mount handle on the opposite side of the knob, facilitating easier setup during experiments.

An in-house-built fixture was designed and developed in the lab to apply linear strain by stretching the nanostructured PDMS sample incrementally along one axis, shown in Figure 1. The fixture applies linear strain via a side-mounted knob that moves the clamps apart linearly. The movement is measured using a linear metric scale with an added Vernier scale for more precise readings of the separation distance. To ensure the sample remains stable during data acquisition, an optical mount handle is attached to the fixture, securing it in place during experiments. Images, spectra, and data were observed and recorded as the sample was strained by the fixture at 500 µm increments. One limitation of the fixture is its ability to hold the samples under high strain (>30%), as the samples tend to release from the clamps, leading to distorted measurements. The morphology, depth, and pitch of the gratings were measured using a Dimension Edge atomic force microscope (AFM, Bruker, San Jose, CA, USA) and analyzed using the Nanoscope Analysis v2.0 software of the AFM. Optical measurements of the grating pitch change while stretching were conducted using a 5 mW He-Ne laser with a wavelength of 632.8 nm. This was carried out by measuring the change between the 0 and ±1 diffraction orders (*m*) used to determine the pitch according to the grating equation (Λ=mλ/sin⁡θ), where Λ is the grating pitch, *λ* is the wavelength of the incident light, and θ is the angle of incidence.

Finally, the PDMS nanostructures were coated with a 40 nm layer of gold for SPR generation and measurement, creating a gold-coated 1 mm thick PDMS slab containing the nanostructures. The resulting SPR spectra were recorded in transmission in air using a spectrometer (Ocean Insight QE Pro, Orlando, FL, USA), with horizontally polarized white light from a Stabilized Halogen Light Source SLS301 (Thorlabs, Newton, NJ, USA) illuminating the gratings at normal incidence as the sample is strained.

## 3. Results

The PDMS samples were positioned in a way to align the gratings’ k-vectors parallel to the direction of strain, ensuring optimal sensitivity to the applied mechanical stress. The overall strain experienced by the sample is quantified as ∆L/L, where *L* represents the original length of the sample.

**Figure 2:**
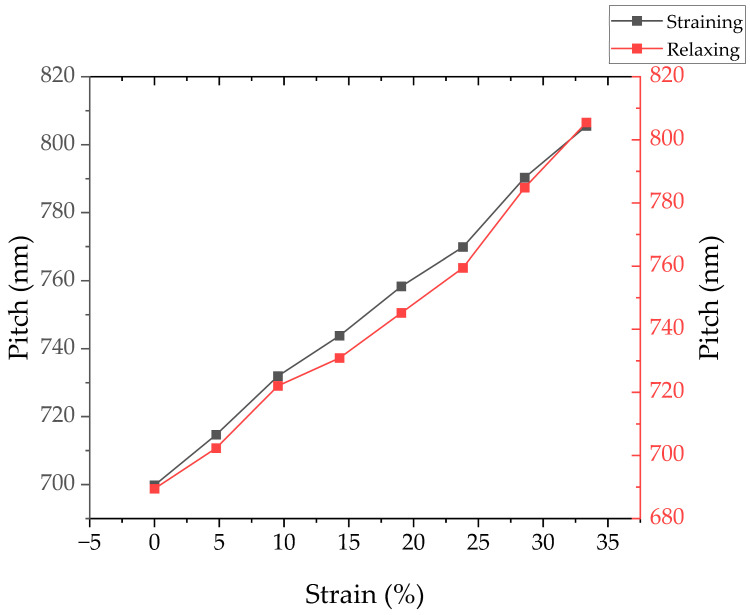
Plot of the change in pitch of the gratings as the PDMS sample undergoes strain and relaxation obtained optically through the change in the non-zero diffraction orders angles. During stretching, the pitch increases by 3.16 nm per percentage of strain applied, while upon relaxation, the pitch alteration is observed to be 3.38 nm per percentage of strain relieved.

To assess the impact of strain on the gratings, the change in pitch is initially evaluated optically through alterations in diffraction order angles. As the sample undergoes stretching, the non-zero diffraction order angles shift in accordance with the pitch variations induced by the applied strain level. These observations are graphically depicted in Figure 2. The PDMS samples had an average length of 14 ± 2 cm, and the maximum strain value corresponds to 18 ± 2 cm of stretching.

Figure 2 illustrates the alterations in pitch as the sample experiences both strain and subsequent relaxation. The initial pitch of the gratings is determined to be a value of 700 ± 1 nm. During stretching, the pitch undergoes a change of 3.2 ± 0.1 nm per percentage of strain applied. Conversely, upon relaxation, the pitch alteration is observed to be 3.4 ± 0.1 nm per percentage of strain relieved. This results in a final pitch of 688 ± 2 nm when the sample returns to its fully relaxed state.

**Figure 3:**
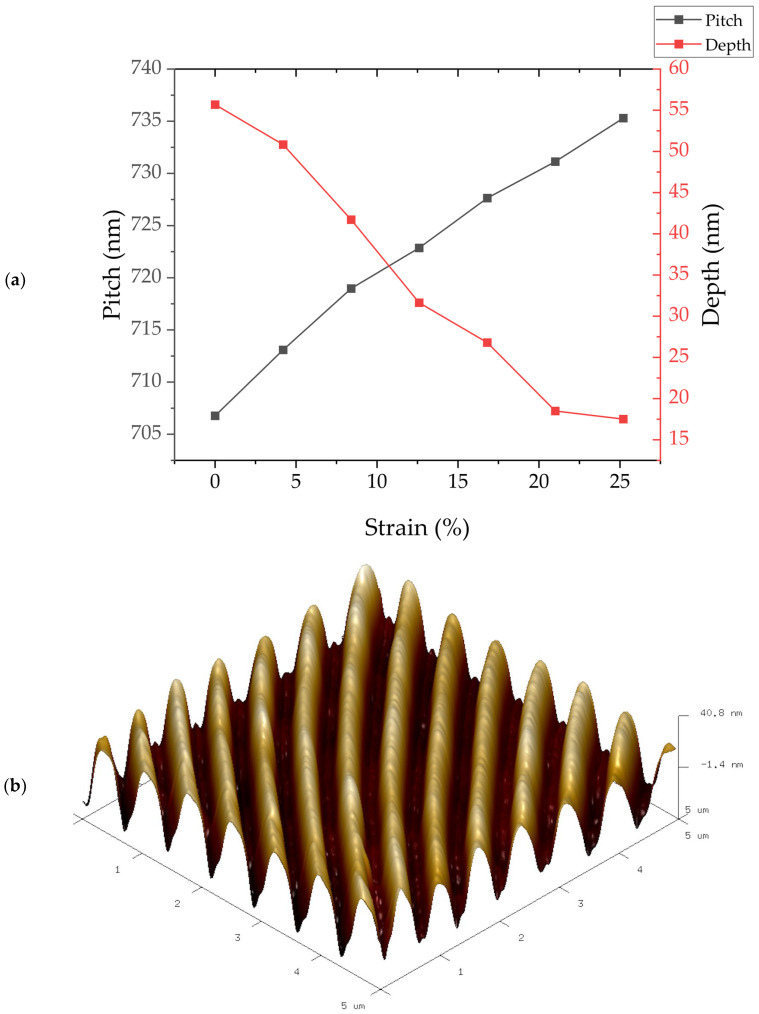
(**a**) Morphological analysis of the gratings using AFM, displaying the average pitch and modulation depth at varying levels of applied strain. (**b**) AFM image obtained at approximately 15% applied strain, illustrating the topographical image obtained of the gratings under mechanical stress.

Morphological analysis was conducted using AFM to gain deeper insights into the pitch and modulation depth changes in the grating as the applied strain level increases. Figure 3a presents the AFM-derived average pitch and modulation depth corresponding to various applied strain values, while Figure 3b showcases an AFM image obtained at approximately 15% applied strain.

The AFM data displayed in Figure 3a confirm the expected increase in pitch with escalating applied strain. Initially, the pitch of the gratings measures 709 ± 1 nm, with a pitch increase of 1.1 ± 0.1 nm per percentage increase in applied strain. In contrast, the initial depth of the gratings is recorded as 56 ± 1 nm, exhibiting a decrease at a rate of 1.7 ± 0.1 nm per percentage of strain. These AFM scans underscore the direct influence of applied strain on the morphology of the gratings, with both pitch and depth properties serving as indicators of strain magnitude.

**Figure 4:**
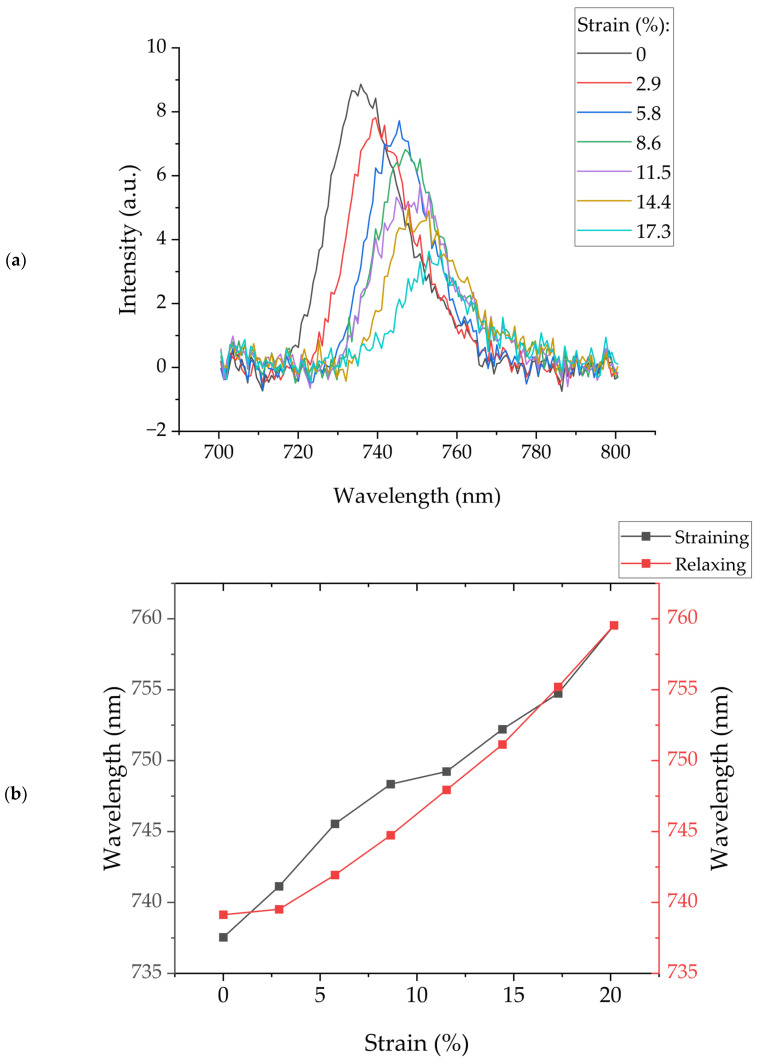
(**a**) Normalized transmission spectra illustrating the plasmonic response of a 40 nm Au-coated PDMS grating as strain increases. The spectra highlight the shift in the SPR peak with applied strain. (**b**) Corresponding shift in SPR wavelength peak as the sample undergoes both strain and relaxation, indicating the sensitivity of the gratings to mechanical stress.

Furthermore, we characterized the plasmonic response of a 40 nm Au-coated PDMS grating in transmission, with air acting as the dielectric medium over the gratings. Figure 4a presents the plotted normalized transmission spectra, showcasing the surface plasmon resonance (SPR) peak and its shift with increasing applied strain, while Figure 4b illustrates the corresponding SPR wavelength peak shift as the sample undergoes strain and subsequent relaxation.

The obtained results from Figure 4b indicate that the SPR peak shifts in a similar fashion as the pitch changes resulting from the AFM. The initial SPR wavelength peak is initially located at 738 ± 1 nm and increases at a rate of 1.0 ± 0.1 nm per % strain applied. Similarly, as the gratings are relaxed, the SPR peak shifts back at a rate of 1.1 ± 0.1 nm per percentage of strain relaxed, ending at an SPR peak wavelength of 737 ± 1 nm with no strain applied.

## 4. Discussion

Upon analyzing optical results, spectral results, and AFM images, it becomes apparent that as the level of one-dimensional strain increases, the grating exhibits an increase in pitch and a decrease in depth. Pitch alterations are indirectly inferred from the diffraction order and the SPR peak, while direct measurements are obtained through in situ AFM imaging under active strain. Similarly, the reduction in depth is directly assessed through the AFM scans, while indirect measurements are derived from the decreasing SPR peak intensity with increasing strain, as seen in Figure 4a. It is well known that a diffraction grating efficiency is directly proportional to its modulation depth. Therefore, as the grating depth decreases with stretching, less light is coupled into the SPR mode, and the SPR peak decreases.

Through the optical diffraction order analysis, a pitch rate change of approximately 3 nm per percentage increase in strain is observed, whereas the AFM and spectral results indicate a rate change of around 1 nm per percentage increase in strain. This disparity can be attributed to differences in the experimental setups. In the diffraction order analysis, where a pitch rate change of approximately 3 nm per percentage increase in strain is noted, the experimental setup utilizes a HeNe laser without focusing lenses. This configuration exposes the sample to a broader area of illumination of approximately 1 mm, resulting in a wider observed diffraction band. Consequently, the diffraction order analysis provides an average measurement of pitch change over this larger measurement area. On the other hand, the AFM and spectral measurements focus on a smaller area of only 500 μm, which constitutes localized regions on the sample. This allows for the detection of subtle changes in pitch at specific points on the grating. As a result, the observed pitch rate change in these measurements, which is approximately 1 nm per percentage increase in strain, reflects the localized surface alterations occurring within these smaller areas.

The disparity in pitch change rates between the diffraction order analysis and the AFM/spectral results underscores the importance of considering the acquisition platform when these nanostructures are used as sensors and how the data are obtained. While the diffraction order analysis offers a broader view of pitch changes across the sample, the AFM and spectral measurements provide insights into localized variations in pitch. Therefore, the data acquisition from these optical strain sensors should be matched to their exact application. Together, these two complementary measurement approaches contribute to a comprehensive understanding of the mechanical behavior of the PDMS gratings under mechanical strain.

**Figure 5:**
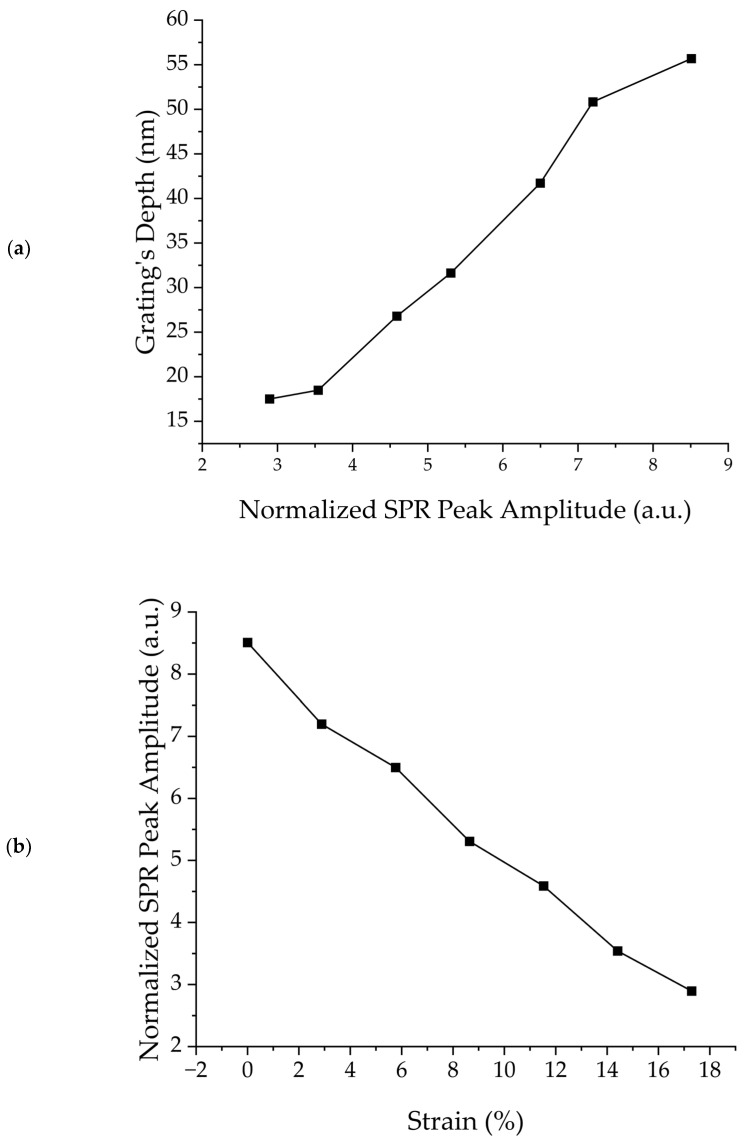
(**a**) Graph illustrating the correlation between the modulation depth of the grating and the intensity of the SPR peak. As the grating depth decreases under strain, the SPR peak intensity exhibits a corresponding reduction. (**b**) Plot demonstrating the decrease in intensity of the SPR peak as a function of strain.

In the context as a SPR strain sensor, the wavelength of the SPR peak serves as an indirect indicator of the grating’s pitch and, consequently, the magnitude of the applied mechanical strain. With a sensitivity of approximately 1.00 nm shift in the SPR peak per percentage increase in strain, this sensor demonstrates a remarkable capability to detect small changes in applied mechanical stress. Notably, despite undergoing stretching to approximately a 20% increase in the original substrate length, the sample exhibits robustness by retaining its surface morphology and its operational integrity upon relaxation. Moreover, the amplitude of the SPR peak serves as another critical parameter closely linked to the applied strain, as illustrated in Figure 5.

Figure 5a displays the relationship between the modulation depth of the grating and the intensity of the SPR peak. As the grating depth decreases under strain, the corresponding SPR peak intensity experiences a proportional reduction. This correlation between grating depth and SPR peak intensity enables a direct means of quantifying strain, as shown in Figure 5b, where the intensity decreases at a rate of −0.32 ± 0.01 a.u. normalized.

## 5. Conclusions

In summary, this study demonstrates the fabrication and characterization of PDMS nanostructures for SPR-based strain sensing. Our results show consistent changes in the pitch and modulation depth of PDMS gratings with increasing strain, indicating the potential of these sensors for real-world applications. Notably, the SPR peak exhibited an increase of approximately 1.0 nm in wavelength and a decrease of approximately 0.3 a.u. in intensity per percentage of applied strain. Furthermore, the capability of the PDMS samples to retain their structural integrity and functionality even after substantial stretching highlights their resilience and suitability for real-world applications requiring flexible and stretchable sensing devices such as tactile sensors for measuring pressure and strain, and as monitoring tools in wearable or prosthetic and orthotic devices. Moreover, the gratings serve as adaptable optical elements capable of manipulating plasmonic and optical properties in response to applied strain [27]. Leveraging the ability to effectively lift-off nanostructures during fabrication and robustness of PDMS, this platform holds potential for the realization of more intricate structures beyond gratings, extending into metasurfaces. This work contributes to the development of flexible sensing technologies, paving the way for future innovations in various fields.

## Data Availability

Dataset available on request from the authors.
